# High prevalence and two dominant host-specific genotypes of *Coxiella burnetii* in U.S. milk

**DOI:** 10.1186/1471-2180-14-41

**Published:** 2014-02-17

**Authors:** Talima Pearson, Heidie M Hornstra, Remy Hilsabeck, Lauren T Gates, Sonora M Olivas, Dawn M Birdsell, Carina M Hall, Sabrina German, James M Cook, Meagan L Seymour, Rachael A Priestley, Ashley V Kondas, Christine L Clark Friedman, Erin P Price, James M Schupp, Cindy M Liu, Lance B Price, Robert F Massung, Gilbert J Kersh, Paul Keim

**Affiliations:** 1 Center for Microbial Genetics and Genomics, Northern Arizona University, Flagstaff, AZ 86011-4073, USA; 2 Rickettsial Zoonoses Branch, Centers for Disease Control and Prevention, Atlanta, GA, USA; 3 Pathogen Genomics Division, Translational Genomics Research Institute, Phoenix, AZ, USA; 4 Current address: Global and Tropical Health Division, Menzies School of Health Research, Tiwi, Northern Territory 0810, Australia

**Keywords:** *Coxiella burnetii*, Q fever, Environmental detection, Genotyping, Phylogeography, Multispacer typing, SNP typing, Canonical SNP, CanSNP

## Abstract

**Background:**

*Coxiella burnetii* causes Q fever in humans and Coxiellosis in animals; symptoms range from general malaise to fever, pneumonia, endocarditis and death. Livestock are a significant source of human infection as they shed *C. burnetii* cells in birth tissues, milk, urine and feces. Although prevalence of *C. burnetii* is high, few Q fever cases are reported in the U.S. and we have a limited understanding of their connectedness due to difficulties in genotyping. Here, we develop canonical SNP genotyping assays to evaluate spatial and temporal relationships among *C. burnetii* environmental samples and compare them across studies. Given the genotypic diversity of historical collections, we hypothesized that the current enzootic of Coxiellosis is caused by multiple circulating genotypes. We collected A) 23 milk samples from a single bovine herd, B) 134 commercial bovine and caprine milk samples from across the U.S., and C) 400 bovine and caprine samples from six milk processing plants over three years.

**Results:**

We detected *C. burnetii* DNA in 96% of samples with no variance over time. We genotyped 88.5% of positive samples; bovine milk contained only a single genotype (ST20) and caprine milk was dominated by a second type (mostly ST8).

**Conclusions:**

The high prevalence and lack of genotypic diversity is consistent with a model of rapid spread and persistence. The segregation of genotypes between host species is indicative of species-specific adaptations or dissemination barriers and may offer insights into the relative lack of human cases and characterizing genotypes.

## Background

*Coxiella burnetii,* the etiological agent of Q fever and a category B biothreat agent, has the potential for rapid, long distance dispersal. This obligate intracellular bacterium is easily aerosolized and has been known to cause infections downwind of a likely source [1,2]. In humans, inhalation is a significant route of infection as 1 to 10 organisms can cause disease [3]. While most cases are relatively mild, some infections result in abortions, premature birth, pneumonia, endocarditis or death. Livestock contaminate the environment by shedding live *C. burnetii* cells in feces, urine and milk; in sheep and goats, birthing tissues contain particularly high quantities of live cells. Viable *C. burnetii* cells can persist in the environment due to resistance to environmental degradation as a small cell variant, however their longevity is unknown. Mild effects of infections in most zoonotic reservoirs enable them to remain ambulatory and facilitate continued transmission; often, domestic and wild animal hosts suffer either no disease, or only mild forms when infected [4].

With the possible exception of New Zealand, *C. burnetii* is found worldwide. Studies of prevalence in livestock have produced highly variable results due to different methodologies and study designs [5], similarly, PCR based detection studies also show variable levels of infection ranging from 20 to 100% of samples [6-10]. Due to the suspected importance of livestock in maintenance and transmission of *C. burnetii*, dairy products have been recently sampled and show high prevalence rates [8,11-13]. Environmental sampling in the United States also shows highly variable but widespread prevalence of *C. burnetii*[14]. In the Netherlands, environmental presence was correlated with incidences of Q fever in humans [15].

With few exceptions, the variability and relatedness among *C. burnetii* detected across the landscape is unknown. As such, we cannot determine the extent to which the current distribution is due to frequent, but isolated occurrences, or a single large outbreak. Despite its ubiquity and importance, genotyping efforts on *C. burnetii* have lagged behind those of other bacterial pathogens because of culturing difficulties and the reliance of genotyping technologies on good quantity/quality DNA obtained through culturing. Previous genotyping methods include: restriction fragment length polymorphisms [16], multiple-locus variable-number tandem-repeat analysis (MLVA) [17,18], multi-spacer sequence typing (MST) [19], representative SNPs derived from MST or whole genome sequences [20,21], and whole genome SNP typing [22]. For *C. burnetii* genotyping, easy and accurate comparisons of results across laboratories are particularly important as they enable the small collections from individual laboratories to be placed into the context of global genotyping efforts.

SNPs derived from MST [20] or whole genome sequence comparisons [21,22] are well suited for inter laboratory comparisons and for sensitive genotyping assays that can inform evolutionary relationships among samples collected from the environment without the need for culturing. In such clonal organisms with no evidence of lateral gene transfer [22], a single SNP allele can accurately define a lineage, allowing for a small subset of loci to be used for genotyping [20,23,24]. PCR assays using TaqMan chemistry have been shown to approach the theoretical minimum level of detection [24,25] and for *C. burnetii*, sensitive detection assays have been developed and used to gauge environmental prevalence. Here, we developed canonical SNP loci into sensitive TaqMan assays and use them for genotyping *C. burnetii* DNA extracted from bovine and caprine milk samples collected from a single herd and from multiple milk processing plants across the USA. We aimed to test whether the current prevalence and distribution of *C. burnetii* is due to the circulation of multiple genotypes which would indicate frequent but unrelated Coxiellosis outbreaks.

## Results

### A single bovine herd

**Figure 1:**
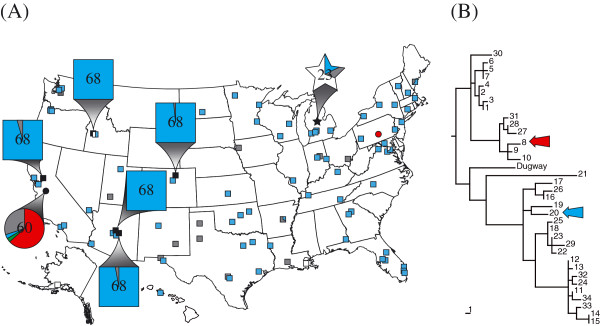
**Phylogeography of samples. (A)** Map shows the location of the sampled Michigan bovine herd (star) and the location of milk processing plants where caprine (circles) and bovine (squares) samples were processed. Expanded shapes indicate the locations of the Michigan bovine herd and the six processing plants from which biweekly samples originated and the total number of samples tested. Expanded shapes also include a pie chart indicating detection and MST genotype results (blue = ST20, red = ST8, green = other unknown ST, grey = unable to genotype, white = negative). **(B)** Phylogenetic tree depicting all known MST genotypes. Colored arrows correspond to STs shown on the map. Tree was drawn according to Hornstra et al. [20] and rooted according to Pearson et al. [22].

In a single herd (n = 120) of dairy cows in Michigan (Figure 1), *C. burnetii* DNA was detected in the milk from 4 of the 20 cows sampled; however, each of the 3 samples collected from the bulk holding tank on the farm were positive (Table 1). Four of these samples contained enough DNA for successful genotyping and the MST genotype was ST20 (Table 1).

**Table 1:** Results for detection and genotyping samples from a single bovine dairy herd

**Sample ID**	**Sample source**	**IS** ***1111 *** result*	**Genotyping result**
M0101	Individual cow	1/9, 39.49	Undetermined
M0100	Individual cow	1/9, 39.50	Undetermined
M0086	Individual cow	1/9, 42.29	Undetermined
M0099	Individual cow	9/9, 31.05	ST20
M0084	Individual cow	Negative	n/a
M0085	Individual cow	Negative	n/a
M0087	Individual cow	Negative	n/a
M0088	Individual cow	Negative	n/a
M0089	Individual cow	Negative	n/a
M0090	Individual cow	Negative	n/a
M0091	Individual cow	Negative	n/a
M0092	Individual cow	Negative	n/a
M0093	Individual cow	Negative	n/a
M0094	Individual cow	Negative	n/a
M0095	Individual cow	Negative	n/a
M0096	Individual cow	Negative	n/a
M0097	Individual cow	Negative	n/a
M0098	Individual cow	Negative	n/a
M0102	Individual cow	Negative	n/a
M0103	Individual cow	Negative	n/a
M0106	Bulk tank	8/9, 36.08	ST20
M0105	Bulk tank	9/9, 35.09	ST20
M0104	Bulk tank	9/9, 35.49	ST20

*Results are given as the total number of replicates that amplified out of nine replicates, followed by the average Ct value for all amplifying replicates.

### Milk processing plants across the USA

Of 134 milk samples bottled at processing plants across 41 states, 95.5% (128/134) tested positive for *C. burnetii* DNA based on the IS*1111* PCR assay [26] (Figure 1 and Table 2). Genotypes from all IS*1111*-positive bovine milk samples were either ST20, were incompletely genotyped (low DNA) but consistent with ST20, or could not be genotyped (Figure 1 and Table 2). Importantly, the ST20-specific SNP locus (Cox56bp10) successfully amplified in 103 samples and all contained the ST20 allele. In the 9 cases where the diagnostic ST20 assay did not amplify, ST20 could not be excluded as none of the other assays implied a different genotype. Only 15 samples had detectable amounts of *C. burnetii* DNA present as determined by IS*1111*, but not enough for SNP genotyping analysis (i.e., all assays failed to amplify either allele). Only six samples tested negative for *C. burnetii* DNA (Table 2). Finally, a sample of caprine milk processed in a plant in Pennsylvania tested positive for *C. burnetii* DNA and had a genotype of ST8 (Figure 1 and Table 2).

**Table 2:** **Geographic distribution and genotyping results of *Coxiella burnetii *** **DNA from commercial milk samples**

**Bottling state**	**Samples**	**Animal source**	**ST20**^***a***^	**Possible ST20**^***b***^	**ST8**^***c***^	**Unable to genotype**^***d***^	**IS** ***1111 *** **negative**
Alabama	1	Bovine	1	0	0	0	0
Alaska	2	Bovine	0	0	0	0	2
Arizona	8	Bovine	7	1	0	0	0
Arkansas	3	Bovine	1	0	0	2	0
California	4	Bovine	4	0	0	0	0
Colorado	3	Bovine	3	0	0	0	0
Connecticut	3	Bovine	3	0	0	0	0
Florida	10	Bovine	10	0	0	0	0
Georgia	3	Bovine	3	0	0	0	0
Hawaii	1	Bovine	1	0	0	0	0
Idaho	2	Bovine	1	0	0	0	1
Illinois	1	Bovine	1	0	0	0	0
Indiana	4	Bovine	3	0	0	1	0
Iowa	1	Bovine	0	0	0	1	0
Kansas	3	Bovine	2	1	0	0	0
Kentucky	2	Bovine	2	0	0	0	0
Louisiana	3	Bovine	1	0	0	1	1
Maine	5	Bovine	3	0	0	2	0
Maryland	4	Bovine	3	0	0	0	1
Massachusetts	1	Bovine	1	0	0	0	0
Michigan	4	Bovine	4	0	0	0	0
Minnesota	5	Bovine	4	1	0	0	0
Nevada	1	Bovine	1	0	0	0	0
New Hampshire	2	Bovine	2	0	0	0	0
New Jersey	2	Bovine	2	0	0	0	0
New Mexico	5	Bovine	4	0	0	1	0
New York	3	Bovine	2	1	0	0	0
North Carolina	1	Bovine	1	0	0	0	0
North Dakota	1	Bovine	1	0	0	0	0
Ohio	1	Bovine	0	0	0	0	1
Oklahoma	6	Bovine	5	1	0	0	0
Oregon	8	Bovine	7	1	0	0	0
Pennsylvania	2	Bovine	1	1	0	0	0
Pennsylvania	1	Caprine	0	0	1	0	0
Tennessee	1	Bovine	1	0	0	0	0
Texas	12	Bovine	6	1	0	5	0
Utah	2	Bovine	1	0	0	1	0
Vermont	3	Bovine	2	1	0	0	0
Virginia	3	Bovine	3	0	0	0	0
Washington	3	Bovine	2	0	0	1	0
Wisconsin	2	Bovine	2	0	0	0	0
Unknown USA	2	Bovine	2	0	0	0	0
TOTAL	134		103	9	1	15	6

^*a*^Samples genotyped as allele G at locus Cox56bp10. This locus is specific to ST20 where allele G indicates ST20 and allele A excludes ST20.

^*b*^Samples failed to be genotyped with the ST20 specific locus Cox56bp10, likely due to low levels of DNA. However, other loci were used to determine groups of likely sequence types and ST20 was not able to be excluded.

^*c*^Samples genotyped as allele C at locus Cox51bp67. This locus is specific to sequence type 8 where allele C indicates ST8 and allele T excludes ST8.

^*d*^All SNP assays failed to amplify either allele.

### Temporal sampling

Using the IS*1111* assay [26], *C. burnetii* DNA was detected in every bovine milk sample (n = 340) representing five commercial brands (each from a different processing plant) that were purchased biweekly (every two weeks) for 32 months (May 2010 through December 2012) (Figure 1 and Table 3). For the bovine milk samples collected across the entire USA, the genotype of all samples was likely to be exclusively ST20. There were 14 samples where the allele for the ST20-specific locus could not be amplified (Table 3), but even in these cases, results from other SNP assays placed the samples in clades that included ST20. Only six samples contained too little DNA for any genotyping.

**Table 3:** **Genotyping results of *Coxiella burnetii *** **DNA from bovine and caprine milk sampled every-other week**

**Brand ID**	**Bottling state**	**Species**	**Samples**	**ST20**^***a***^	**Possible ST20**^***b***^	**ST8**^***c***^	**Possible ST8**^***d***^	**Other STs**	**Unable to genotype**^***e***^
A	Arizona	Bovine	68	67	1	0	0	0	0
B	Arizona	Bovine	68	63	3	0	0	0	2
C	California	Bovine	68	59	6	0	0	0	3
D	Colorado	Bovine	68	65	2	0	0	0	1
E	Idaho	Bovine	68	66	2	0	0	0	0
F	California	Caprine	60	2	0	28	10	2	18
		TOTALS	400	322	14	28	10	2	24

^*a*^Samples genotyped as allele G at locus Cox56bp10. This locus is specific to sequence type 20 where allele G indicates ST20 and allele A excludes ST20.

^*b*^Samples failed to be genotyped with the ST20 specific locus Cox56bp10, likely due to low levels of DNA. However, other loci were used to determine groups of likely sequence types and ST20 was never excluded.

^*c*^Samples genotyped as allele C at locus Cox51bp67. This locus is specific to sequence type 8 where allele C indicates ST8 and allele T excludes ST8.

^*d*^Samples failed to be genotyped with the ST8 specific locus Cox51bp67, likely due to low levels of DNA. However, other loci were used to determine groups of likely sequence types and ST8 was never excluded.

^*e*^All SNP assays failed to amplify either allele.

Caprine milk from a single brand and processing plant was also sampled biweekly for 28 months (September 2010 through December 2012). Like the bovine milk, these 60 samples all tested positive for *C. burnetii* DNA; however, only 2 samples were ST20 (Figure 1 and Table 3). Of the caprine samples, 28 were ST8 and ten were likely ST8. Two samples were neither ST8 nor ST20, however low DNA concentrations did not allow determination of the exact sequence type (Table 3).

## Discussion

Our results show that the current distribution of *C. burnetii* is the result of a few highly fit clones that appear to be largely confined to individual livestock species. The concept of distinct clades associated with species specific restrictions may explain the low apparent rate of clinical disease among human populations despite the high prevalence of these bacteria. Among our samples, two sequence types were highly prevalent: ST8 was exclusively found in samples from goats while ST20 dominated cow’s milk with only two examples of ST20 from goats. This pattern is consistent with other smaller studies where likely ST20 isolates (see below) were from cattle [21,27,28] and rarely from goats: a single ST20 sample attributed to a goat in France [21] and abortions in a large commercial dairy goat herd in the UK [29]. Likewise, recent ST8 samples have been collected from sheep, goats and humans [21,27,30,31]. This tendency for host restriction may be the result of a stochastic introduction into a large livestock population allowing for an increase in frequency, spread through trade, but constrained to that population through anthropogenic isolation of livestock species. However, as both genotypes show a tendency for host restriction and similar patterns are found in Europe [21,27,28,30] as well as the USA, it seems more likely that these genotypes are evolutionarily adapted to certain host species.

Genotyping historical collections of *C. burnetii* has provided a baseline for environmental distribution of sequence types [17,19,20,32]. Interestingly, contemporary sampling yields only a small subset of the known genotypes, many of which are found across multiple studies [21,27,28,30] (Kersh et al., Genotypes of *Coxiella burnetii* strains found in the United States environment, 2006-2008, in preparation). In some cases, subtypes of the same MST genotypes were identified [27,30,33]. Consistent with these findings, our genotyping of milk samples revealed only three or four MST genotypes; while only two samples had unknown genotypes (and may both have the same genotype), the genotypes of all other samples are likely to be either ST20 or ST8. It is important to note that additional genotypes not detected by our sampling may be circulating at very low levels. A high proportion of recent milk, placenta, and mucus samples from goat, cow and sheep farms in Spain were ST20, but none were ST8 [27]. Kersh et al. recently genotyped *C. burnetii* DNA from US environmental samples and found ST8, ST16/26, and ST20 genotypes. Samples associated with goats were ST8 and all ST20 samples came from cattle dairies (Kersh et al., Genotypes of *Coxiella burnetii* strains found in the United States environment, 2006-2008, in preparation). In the Netherlands, a study by Tilburg et al. [28] sampled ST20 from cattle and ST33 from humans, sheep and goats. Huijsmans et al. [21] also genotyped recent samples from the Netherlands, albeit not with MST. However, overlapping reference samples, the results from Tilburg et al. [28] and a comparison to the phylogenetic relationships of MST genotypes, suggests that the Huijsmans [21] genotypes 1, 2, 4, 6 and 8 are likely to be (or be closely related to) MST genotypes ST33, ST20, ST20, ST8 and ST18 respectively.

While likely ST8 samples have been associated with recent livestock and human clinical samples, such associations with likely ST20 samples are rare (for example see [29]) and it is not clear if any of the Spanish ST20 samples were from animals with clinical manifestations [21,27,28,30]. From the recent outbreak in a UK dairy goat herd [29] and historical collections, it is clear that ST20 can cause disease in humans and livestock [19,20]. The scarcity of ST20 among clinical samples, despite being the dominant genotype among cow milk samples, suggests that U.S. ST20 strains have a reduced ability to cause disease in humans or cause a very mild form.

Prevalence of *C. burnetii* on goat and cow farms has been previously assessed, but comparisons across studies are difficult due to different serological or DNA-based detection methods. Sampling individual animals, herds, or products pooled across herds also confounds comparisons although as expected, prevalence generally increases as bulk samples become inclusive of more individuals [6,8,13,34-37]. Similarly, we found that milk from four of 20 sampled cows were positive while all 3 samples from the bulk milk holding tank (containing milk from 120 cows) were positive. Our milk samples from retail brands bottled in commercial processing plants likely include milk pooled from different (and much larger) dairy farms, making it impossible to know the extent and distribution of infections among cows and herds. However, our detection of *C. burnetii* DNA in every goat and cow milk sample from the same brands (i.e. processing plants) over time and >95% of milk samples from processing plants across the USA shows high prevalence at either or both the individual and herd levels. Indeed, the prevalence rate reported here is comparable to the high rates reported in other studies [8,12,13]. Notwithstanding existing immunity, infectious diseases are density dependent, leading us to suspect that the ratio of infected to uninfected cows on some farms may be greater than our single farm results. Nonetheless, while a small number of infected animals may contaminate a large quantity of milk, it is probable that a significant portion of the 9.2 million dairy cows in the USA [38] are infected with *C. burnetii* at any given time [13].

Across the ~2.5 year period of sample collection, there was no variation in prevalence of *C. burnetii* DNA in milk samples and almost no variation in genotypes detected. As processing plants receive milk from the same dairies over time, it is likely that the same herds and even the same animals were sampled multiple times. Major temporal changes in prevalence and genotypes should be detectable. Indeed, minor genotypes were detected among the goat milk samples, indicating ephemeral emergence of different types. Conversely, subtle changes may be masked by the milk pooling process and the ability of a single infected animal to contaminate large quantities of milk. Indeed, other studies suggest that there is evidence of seasonality: In cows, shedding in milk is not associated with parturition [39] although seroprevalence is highest in the Autumn [40]. In goats, *C. burnetii* are highly abundant (up to 10^9^ organisms/g of placental tissue) in birth tissues [41] and more likely to be shed after parturition [42]. Human infections are therefore likely to be more common during livestock birthing seasons [43], suggesting that infection variation among goat herds might also be seasonally linked. Seasonality is often associated with a boom and bust cycle of transmission, and the lack of strong seasonal patterns may increase disease persistence.

As pathogens are dispersed across the landscape, elapsed time allows for cellular replication and opportunities for genetic mutations to accumulate, providing genetic signatures to identify the patterns and speed of dissemination. The presence of the same genotypes among samples from across the country and the world is indicative of rapid dispersal of particular gentoypes and subsequent ecological establishment across these regions. While a paucity of historical samples and sampling efforts prevents us from estimating when these STs became dominant, no ST20 isolates were collected in the U.S. before 2007 [20]. Interestingly, the only U.S. *C. burnetii* samples isolated from milk with a known date were obtained from cows in California (1947) and Ohio (1958) [20]. Both samples are ST16/26, showing that the dominant genotype among cows may have recently changed. Higher resolution genotyping will be important for discerning dissemination patterns and mechanisms of these *C. burnetii* genotypes as dispersal may be due to long distance aerosol spread, trade, or other anthropogenic means. For example, sexual transmission through semen [44] from the small stock of infected breeding bulls used to breed Holstein cows throughout the world could result in shared genotypes. However, additional resolution among ST20 and ST8 samples has been shown with MLVA [27] and demonstrates that dissemination speed and patterns may have allowed for the accumulation of genetic differences and thus discerning patterns, mechanisms and barriers to dispersal may be possible. Unfortunately, MLVA is not as well suited for genotyping low levels of target DNA and the little additional resolution afforded by MLVA is not likely to be sufficient for defining detailed patterns of dispersal for this pathogen. High infectivity, low virulence and ease of aerosolization coupled with the speed and global reach of modern trade has likely resulted in these complex and subtle patterns of dissemination that will be challenging to resolve. Whole genome sequencing will likely provide additional signatures that may prove to be our best hope for maximizing genetic resolution, untangling dispersal patterns and better estimating the speed and mechanisms of dispersal for *C. burnetii*.

## Conclusions

*Coxiella burnetii* is a highly infectious and easily aerosolized biothreat agent that is abundant in the environment and among livestock, yet few human Q fever cases are reported. Despite high potential for human infections, knowledge of phylogeographical patterns are lacking due to difficulties in culturing this obligate, intracellular bacterium. Using sequences from diverse strains, we developed and employed a genotyping system that does not require culturing and is capable of genotyping residual *C. burnetii* DNA from pasteurized milk. Our results show very high prevalence of two dominant genotypes, one for bovine milk and one for caprine milk, likely due to rapid population expansion and persistence among U.S. livestock. Different dominant genotypes associated with different host species indicate barriers to cross-species transmission and may explain why we have not seen an associated proliferation of human infections. The genetic patterns coupled with spatial analysis suggest independent co-circulation of multiple *C. burnetii* genotypes among different dairy livestock species in the United States.

## Methods

Assays designed based on SNP signatures are ideal for genotyping. Real-time PCR assays incorporating TaqMan chemistry are highly sensitive and can thus be used for detection and genotyping of DNA from environmental samples without culturing. The IS*1111* detection assay [26] is particularly sensitive due to the presence of multiple target copies in *C. burnetii* genomes, however single target SNP genotyping assays amplified in 92.1% of IS*1111* positive samples (493/535). Genotype information from SNP assays are easy to score and unambiguous. The genotyping assays used here are based on signatures derived from MST [19], and presented by Hornstra et al. [20], allowing the results to be directly compared to previous MST based genotyping work without shared reference samples. Single copy SNP alleles in *C. burnetii* are evolutionarily stable, reducing the likelihood of evolutionary convergence [22]. Once a mutation occurs, every descendant and no unrelated isolates can be expected to share that allele. For genotyping, this means that a single SNP assay can define a clade, and even when some assays fail to amplify due to low concentrations of target DNA, phylogenetic placement of the sample at varying hierarchical phylogenetic levels is possible.

### Samples and DNA extraction

With the exception of samples from individual cows from a herd in Michigan, all samples were purchased from grocery stores around the country. We determined the location of the processing plant for each sample from the code on the packaging that indicates the processing plant. Most milk samples were either fresh or frozen at −20°C before DNA extraction. Unpasteurized samples were autoclaved at 104°C for 20 minutes before being processed. IACUC oversight for all samples (including sampling individual cows) was not required.

DNA was extracted in triplicate for each sample using a proteinase K and chelex protocol (see Additional file 1). For each sample, a no template control (NTC) was also included in the DNA extraction protocol to detect any cross-contamination. The quality of the DNA extractions were assessed by running a generalized 16S rRNA assay [45] on each extraction to ensure that PCR quality DNA was obtained. Any samples that failed 16S rRNA quality controls were re-extracted.

### Detection and genotyping of *C. burnetii* DNA

*C. burnetii* DNA was detected in samples using an assay designed to detect the multicopy IS*1111* element [26]. For each DNA extract, this assay was run in triplicate with each of the three DNA extraction replicates and the extraction NTC. If the extraction NTC amplified, the sample was put through the extraction protocol again. If any of the nine extract replicates amplified, the sample was considered to be positive for *C. burnetii* DNA.

Samples that were positive for *C. burnetii* DNA were genotyped with TaqMan assays derived from signatures presented by Hornstra et al. [20]. Primer and probe designs as well as reaction conditions are included in Additional file 1. For each PCR, an additional NTC was included to help detect cross contamination during template addition. Cross contamination was a concern as the genotype results from most samples were identical. It is important to note also that before genotyping these samples, we had not had any samples of ST20 in our laboratory. To further ensure the integrity of positive PCR results and that shared genotypes across samples were not due to contamination from a positive control, we designed synthetic positive controls for each assay containing *C. burnetii* signatures as well as a non-bacterial sequence targeted by a probe with a different dye color (Additional file 1).

## Competing interests

The authors declare that they have no competing interests.

## Authors’ contributions

HMH, RH, LTG, SMO, CMH, SG, JMC, MLS, RAP, AVK, CLCF, EPP carried out sample collection, sample processing, and genotyping. HMH, RH, LTG, SMO, DMB, CML, LBP participated in assay and synthetic positive control design and validation. TP, HMH, JMS, RFM, GJK, PK conceived of the study and participated in its design and coordination. TP, HMH, RFM, GJK, PK drafted the manuscript. All authors read and approved the final manuscript.

## Supplementary Material

Additional file 1 **Specific information pertaining to the treatment and storage of milk samples used in the study, DNA extraction methods used on the milk samples, and assay information including primer and probe sequences as well as PCR conditions.** Also included are the methods for constructing self-reporting, synthetic positive control templates. Click here for file
