# Supplementary material for: High prevalence and two dominant host-specific genotypes of Coxiella burnetii in U.S. milk
Source: BMC Microbiol. 2014 Feb 17;14:41. doi: 10.1186/1471-2180-14-41 (PMC3936997; doi:10.1186/1471-2180-14-41)
Supplement: Additional file 1 — Specific information pertaining to the treatment and storage of milk samples used in the study, DNA extraction methods used on the milk samples, and assay information including primer and probe sequences as well as PCR conditions. Also included are the methods for constructing self-reporting, synthetic positive control templates. [file 1471-2180-14-41-S1.pdf]

Table of Contents
I. DNA Extraction
II. Assay Information
III. Genotyping
IV. Self-reporting Synthetic Positive Control
V. References
VI. Figures
VII. Tables

I. DNA Extraction

This section describes the methods used for extracting *Coxiella burnetii* DNA from milk samples for the purpose of genotyping using PCR-based analyses. Due to the techniques used in this protocol and the often minimal amount of *C. burnetii* DNA present in each sample, we generated three extractions per sample.

Milk samples were mixed well by inversion or vortexing and 3 x 500 µL aliquots were placed into individually labeled 1.5 mL microcentrifuge tubes. Additionally, 500 µL of sterile water was added to a fourth microcentrifuge tube to serve as an extraction control. Twenty microliters of Proteinase K Solution (Life Technologies Corp., USA; p/n AM2546) were added to each tube. Additional Proteinase K Solution (up to 40 µL total) was added to tubes where the milk sample appeared spoiled or contained chunks. Samples were mixed by inversion or vortexing and then incubated at 55°C for 60 minutes. Post incubation, tubes were again mixed and then centrifuged at 14,000 rpm for 5 minutes. Centrifugation resulted in the samples separating into a lipid layer, whey, and residual solids. We removed the lipid layer and supernatant (whey) by quickly inverting the tubes over a waste container being careful not to disturb the pellet. A sterile, cotton-tipped swab was then used to dry out the inside of the tube and to remove any remaining lipids that may have adhered to the inside of the tube. Five-hundred microliters of a 5% Chelex 100 Resin (Bio-Rad Laboratories, USA; p/n 142-1253) solution were added to each tube and tubes were incubated at 95-100°C for 20 minutes. Post incubation, the tubes were allowed to cool to room temperature and then centrifuged at 14,000 rpm for 5 minutes. Post-centrifugation, the tubes were handled carefully as the chelex solution does not form a compact pellet. The supernatant containing the DNA was removed with a pipette and transferred to a new, sterile, 1.5 mL microcentrifuge tube. DNA extractions were stored at -20°C (short-term) or -80°C (long-term) until use.

II. Assay Information

We developed ten of the single nucleotide polymorphism (SNP) loci from Hornstra et al. (2011) into TaqMan® dual-probe genotyping assays (Supplemental Table 1). When specifically genotyping low levels of target DNA, the sequence specific primer and allele-specific probe design of a TaqMan® dual-probe assay typically allows for less ambiguous SNP calling when compared to a SYBR-MAMA or melt-MAMA assay, in our experience.

Each TaqMan® assay we designed targets a polymorphic locus in the *C. burnetii* genome that was found to be specific to either a clade of *C. burnetii* multi locus spacer typing (MST) genotypes or a single MST genotype (Glazunova et al., 2005; Hornstra et al., 2011). Supplemental Figure 1 shows the phylogenetic placement of each SNP locus including the nucleotide for the ancestral and derived alleles for each locus for the assays presented here. In addition, for some of our assays we also designed a self-reporting synthetic positive control (see Section IV and Supplemental Table 2).

III. Genotyping

Our TaqMan® dual-probe assays were used to genotype DNA extractions from milk that were determined to be free of PCR inhibitors via a generalized 16S rRNA assay (Liu et al., 2012) and also positive for *C. burnetii* DNA via the IS1111 assay (Loftis et al., 2006). For each assay (Supplemental Table 1), 1 µL of DNA was used in a total reaction volume of 10 µL that contained 5 µL of 2x TaqMan® Universal PCR Master Mix (Life Technologies, CA, USA; p/n 4304437), 0.45 µL of each 20 µM primer, 0.10 µL of each 20 µM MGB probe, and 2.9 µL of sterile, molecular grade water. If synthetic positive controls were used (see section IV) than additionally, 0.10 µL of a 20 µM universal NED MGB probe (NED-CGACTGTGTTAGGTCAC) were added and the amount of water was adjusted to 2.8 µL.

Thermal cycling conditions were: 50°C for 2 min., 95°C for 10 min., followed by 45 cycles of 95°C for 15 sec., and 60°C for 1 min. and were performed on an Applied Biosystems 7900HT Fast real-time PCR system with SDS v2.4 software. Allele calls were used in conjunction with Supplemental Figure 1 to determine a sample's placement into a clade of genotypes or a specific genotype.

IV. Self-reporting Synthetic Positive Control

Real-time PCR TaqMan® dual-probe assays require the inclusion of two positive controls, one per allele SNP state, with every experiment. With this, there is always a risk of reporting false positive results from samples due to possible cross contamination with positive controls. When assays are being used for detection or genotyping of low-levels of target DNA it can be particularly difficult to differentiate cross contamination from true signal as cross contamination from positive controls often presents as a weak signal. To effectively identify false signals, we present a generic molecular approach for generating a synthetic positive control designed to self-report its synthetic status during real-time PCR.

Design. Two synthetic positive controls, one per allele SNP state, must be generated for every unique assay. Each allele-specific synthetic positive control is constructed by two separate, hierarchically ordered, PCR processes (Supplemental Figure 2) that produces a final synthetic template which contains the sequences to the following targets per unique assay: forward primer sequence, universal synthetic sequence (CGACTGTGTTAGGTCAC), allele-specific SNP containing probe sequence, and reverse primer sequence. The purpose of the first PCR is to generate an intermediate template that contains the following targets: allele-specific SNP

containing probe sequence, synthetic sequence, and reverse primer sequence. Genomic DNA (gDNA) is used as the template in the first PCR (Supplemental Figure 2A). The product amplification is generated by a reverse primer and a customized forward primer where the 3'end targets the SNP allele sequence on the gDNA and the 5'end carries the synthetic sequence. The final PCR product from this reaction serves as the template for the second PCR (Supplemental Figure 2B). The second PCR (Supplemental Figure 2B) incorporates the forward primer sequence of the assay onto the intermediate template. This is accomplished by amplifying a product with a reverse primer and a customized forward primer where the 3'end targets the synthetic sequence on the intermediate templates and the 5'end carries the assay's forward primer sequence. This final PCR amplicon from the second PCR is used as the synthetic positive control template for their unique assay. The internal synthetic sequence is targeted by a NED-labeled TaqMan® probe containing the corresponding sequence. Increased NED fluorescence over the course of the real-time PCR run confirms the success of the PCR reaction while simultaneously revealing the synthetic status of the positive control.

Allele-specific synthetic positive controls. The allele SNP-state of the synthetic positive control is determined by the base identity of the 3' end of the forward primer used in the first PCR process. The single 3'end mismatch between a forward allele-specific primer and its non-allelic genomic template does not measurably impede the PCR amplification of product (Supplemental Figure 2A). As a consequence, the same starting genomic DNA template can generate synthetic positive controls possessing either SNP states.

PCR conditions. Each PCR process was performed using conventional PCR master mix composed of 300 nM each of a forward allele-specific primer and a common reverse primer (IDT, CA, USA), 1x PCR buffer without $MgCl_2$ (Life Technologies, CA, USA), 2 mM $MgCl_2$ (Life Technologies, CA, USA), 200 µM of each dNTPs (Life Technologies, CA, USA), 0.8 units of Platinum *Taq* DNA polymerase (Life Technologies, CA, USA), 1 µl of template at ~1ng/µl or 1/100,000 dilution of intermediate PCR product, and molecular grade water to a final volume of 10 µl. PCR amplifications were conducted on an MJ Research DNA engines 96 well block thermal cycler equipped with hot bonnets. PCRs were raised to 94°C for 5 min to denature the DNA and activate the *Taq* DNA polymerase, then cycled (total of 35 times) at 94°C for 30 sec, 55°C for 30 sec, 72°C for 30 sec, with a final extension at 72°C for 5 min.

Utility. To construct our synthetic positive template controls (synPTCs) for this study, we used genomic material from *C. burnetii* strain 9 Mi phase II c4, the primer sets in Supplemental Table 2, and the PCR conditions listed above. While this method of constructing synPTCs worked well for many of our assays we found that for one assay, Cox56bp10, the synPTCs degraded over time such that the NED signal was no longer present in our amplification reactions. Unfortunately, this negated their utility as a self-reporting positive control. We found that one solution to this issue was to purchase two oligos (see Supplemental Table 1) that contained the exact sequence that theoretically our synPTCs for Cox56bp10 would have if we had constructed them in house. While ordering two longer oligos was more expensive than ordering four shorter ones, we have found the longer ones to be more stable and to more

consistently produce a suitable NED signal. Despite this, synthetic PTCs constructed for other assays in this study and for use in similar work with *Francisella tularensis* have not degraded over time and consistently produce good results (D. Birdsell, unpub.) which suggests that the stability of constructed synPTCs may be dependent on the exact sequence of the nucleotides being targeted.

V. References

Glazunova, O., V. Roux, O. Freylikman, Z. Sekeyova, G. Fournous, J. Tyczka, N. Tokarevich, E. Kovacava, T. J. Marrie, and D. Raoult. 2005. *Coxiella burnetii* genotyping. Emerg Infect Dis 11:1211-1217.

Hornstra, H. M., R. A. Priestley, S. M. Georgia, S. Kachur, D. N. Birdsell, R. Hilsabeck, L. T. Gates, J. E. Samuel, R. A. Heinzen, G. J. Kersh, P. Keim, R. F. Massung, and T. Pearson. 2011. Rapid Typing of *Coxiella burnetii*. PLoS One 6:e26201.

Liu, C. M., M. Aziz, S. Kachur, P. R. Hsueh, Y. T. Huang, P. Keim, and L. B. Price. 2012. BactQuant: an enhanced broad-coverage bacterial quantitative real-time PCR assay. BMC Microbiol 12:56.

Loftis, A. D., W. K. Reeves, D. E. Szumlas, M. M. Abbassy, I. M. Helmy, J. R. Moriarity, and G. A. Dasch. 2006. Rickettsial agents in Egyptian ticks collected from domestic animals. Exp Appl Acarol 40:67-81.

Pearson, T., H. M. Hornstra, J. W. Sahl, S. Schaack, J. M. Schupp, S. M. Beckstrom-Sternberg, M. W. O’Neill, R. A. Priestley, M. D. Champion, J. S. Beckstrom-Sternberg, G. J. Kersch, J. E. Samuel, R. F. Massung, and P. Keim. 2013. When outgroups fail; phylogenomics of rooting the emerging pathogen, *Coxiella burnetii*. Syst Biol 62(5):752-762.

VI. Figures

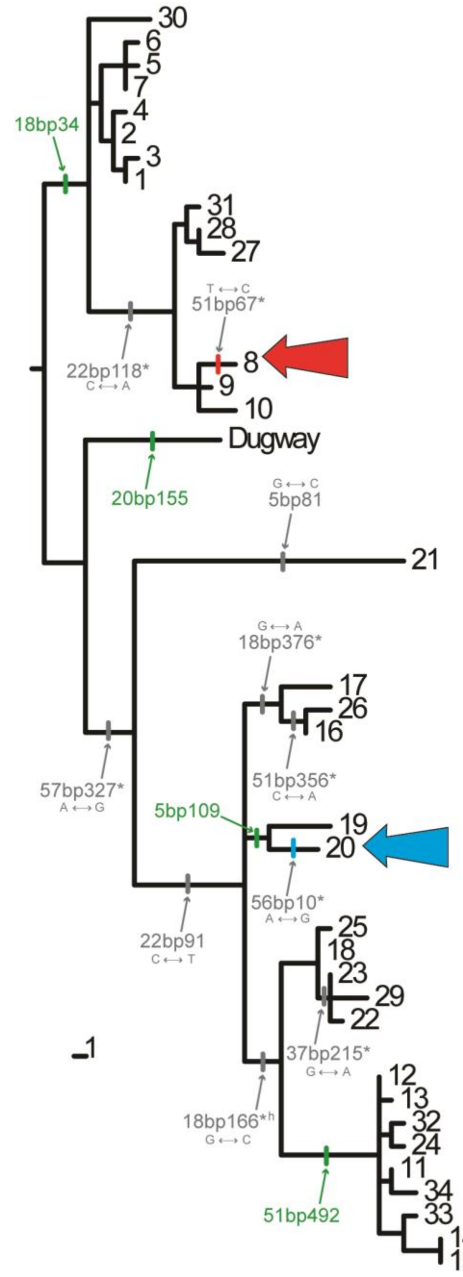

Supplemental Figure 1. A rooted, SNP-based phylogenetic tree for *Coxiella burnetii* multi-locus spacer typing genotypes. Each genotype from Glazunova et al. (2005) and Hornstra et al. (2011) are shown at the ends of the branches. Canonical SNP loci are positioned at the midpoint of the branch leading to the clade or genotype that each locus defines. The ten loci targeted in this study and tested using a TaqMan design are shown in gray with the ancestral and derived allele states for each locus, based on Pearson et al. (2013), shown with the locus name. The ancestral allele state is to the left of the double arrow and the derived allele state is to the right. An (*) indicates the availability of a melt-MAMA assay in Hornstra et al. (2011), (h) denotes the assay that had an incidence of homoplasy (Hornstra et al., 2011). Additional loci presented in Hornstra et al. (2011) that have not been converted to TaqMan assays but are available in a melt-MAMA format are shown in green. Large, colored arrows point to the two genotypes that were most prevalent in this study: ST8 in goat's milk (red arrow) and ST20 in cow's milk (blue arrow).

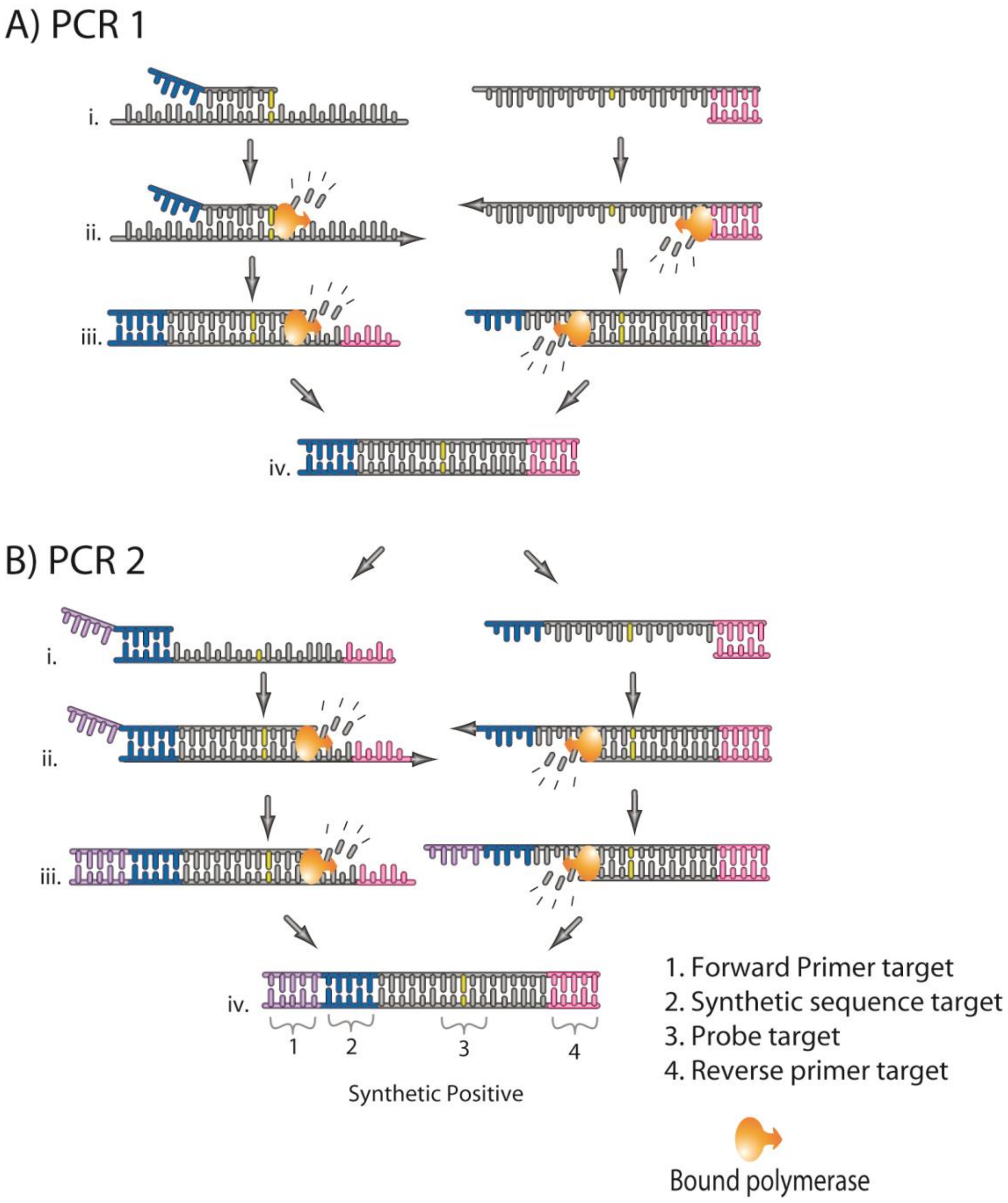

Supplemental Figure 2. The principle of the synthetic positive control design. This scheme shows two separate PCR processes used in a step-wise fashion to generate a single synthetic positive control (A & B). The annealing of the allele-specific (AS)-forward primer and reverse primer to the genomic DNA template is shown with the forward primer labeled with a synthetic sequence at the 5'end (Ai). (Aii) *Taq* Polymerase extends from the 3' end AS-forward and reverse primers. The second PCR cycle replicates from a newly synthesized DNA template made in the previous step (Aiii) and the resulting product contains the synthetic sequence, SNP containing probe sequence, and reverse primer sequence (Aiv). This amplicon serves as the template for the second PCR process (Bi). The amplicon generated by the second PCR process serves as the final synthetic positive control (Biv). This positive control contains the following targets: Forward primer sequence, reverse primer sequence, synthetic sequence, and allele-specific SNP containing probe sequence.

VII. Tables

Supplemental Table 1. Sequence information for ten *Coxiella burnetii* genotyping assays.

Branch[a]	Assay Name[a]	Base Position in RSA493 (GenBank: AE016828.2)	Base Call (ancestral/ derived)	Primer, Probe, and Synthetic Positive Control Sequences (5'→3')[b]	
Br.I/II/III	Cox22bp91	378,762	C/T	Fwd:	GCCTTCCATTATTGATTTATTGCCAAGA
				Rev:	GCAAAAAGTGAGTGATAGCTGAGGAA
				Anc:	VIC-TGGTGCTCCCcTGTAGTG
				Der:	6FAM-TGGTGCTCCCtTGTAGTG
Br.I.001	Cox18bp376	283,398	G/A[c]	Fwd:	CTTAAGTTGGCGCTTCTGTGATT
				Rev:	GTCTCGGTTTAAATCAAGTAAGGGTAGT
				Anc:	VIC-CCCTTTTTTTGgCCTTCGT
				Der:	6FAM-CCCTTTTTTTGaCCTTCGT
Br.I.003	Cox51bp356	824,910	C/A	Fwd:	CGGAAATTTCACACGTATCTGCTAA
				Rev:	ACCATTGCGAGCTTATCATCGATTT
				Anc:	6FAM-TAGCAATAACAcATTTCG
				Der:	VIC-TAGCAATAACAaATTTCG
Br.II.001	Cox18bp166[d]	278,908	G/C	Fwd:	GCGAGGCCGTCTGCTGTA
				Rev:	AGAGAGCGAGGTAAAGAGGCA
				Anc:	6FAM-CAAAACCTTCACGgCC
				Der:	VIC-AAAACCTTCACGcCC
Br.II.004	Cox37bp215	657,611	G/A[c]	Fwd:	GCTGGGTGAAAAATTAATTCGGATTCA
				Rev:	AGGAAATTTCTGGTTAAACCTTTCAAGGA
				Anc:	6FAM-TTTATCTGGGAgCTTTTA
				Der:	VIC-AGTTTATCTGGGAaCTTTTA
Br.III.003	Cox56bp10	886,387	A/G	Fwd:	ACTCCCGGCTCTGATTATGGAA
				Rev:	CCAAGCTCTCTGTGCCCAAT
				Anc:	6FAM-CCACAGCTaCTGTTGC
				Der:	VIC-CCACAGCTgCTGTTGC
				SynAnc:	ACTCCCGGCTCTGATTATGGAAggatcgactgtgttaggtcacATAGTCTTAGCTCTGATTGCAACAGtAGCTGTGGG<br>ATTGGGCACAGAGAGCTTGG
				SynDer:	ACTCCCGGCTCTGATTATGGAAggatcgactgtgttaggtcacATAGTCTTAGCTCTGATTGCAACAGcAGCTGTGGG<br>ATTGGGCACAGAGAGCTTGG
Br.IV/VI	Cox57bp327	893,096	A/G	Fwd:	ACGTTGGACTACTATTACTTGTCGGATA
				Rev:	GCTAATCAGTTAGTCAGATATCTTTAATTTTAATCGGT
				Anc:	VIC-TTTGCCGACTTaATTAG
				Der:	6FAM-TTTGCCGACTTgATTAG

Supplemental Table 1. cont.

Branch[a]	Assay Name[a]	Base Position in RSA493 (GenBank: AE016828.2)	Base Call (ancestral/ derived)	Primer, Probe, and Synthetic Positive Control Sequences (5'→3')[b]	
Br.IV.011	Cox22bp118	378,789	C/A	Fwd:	GCTGTCGGGTGGTGCT
				Rev:	GCGCTAAGCAAAAAGTGAGTGATAG
				Anc:	6FAM-ATTGAACGAcAATTAC
				Der:	VIC-ATTGAACGAaAATTAC
Br.IV.015	Cox51bp67[e]	824,623	T/C	Fwd:	TCGAAAAATACAGCAGCTCTCAAGT
				Rev:	CCAAAGAGTTGAGCGAAGAAAAAGAAA
				Anc:	6FAM-TTTACTAATTTtTCaTAATCTT
				Der:	VIC-TACTAATTTcTCgTAATCTT
Br.V.001	Cox5bp81	77,587	G/C	Fwd:	CGAGGTGTTTGGTGTGTTGAA
				Rev:	GGAGAGGGACAATACGTGCTTATG
				Anc:	6FAM-TTCGCAgTGATATGC
				Der:	VIC-CTAGTAATTTCGCAcTGATATGC

[a]Branch and assay designation from Hornstra et al., (2011).

[b]Fwd, sequence for the forward primer; Rev, sequence for the reverse primer; Anc, sequence (including florescent dye label) for the MGB probe that is specific to the ancestral allele; Der, sequence (including florescent dye label) for the MGB probe that is specific to the derived allele; SynAnc, sequence for the synthetic positive control template for the ancestral allele; SynDer, sequence for the synthetic positive control template for the derived allele. For each probe sequence, the target SNP is shown in lower-case and red text. If additional nucleotide differences are present between two probes in a single assay they are given in lower-case green text. For the synthetic positive control sequences, the target SNP is also shown in lower-case and red text; the target region for the allele-specific probe is double-underlined. Non-native sequence in the synthetic template control is shown in blue lower-case text; the region specific to the synthetic template specific probe is underlined with a single line.

[c]Assay was designed from the reverse compliment.

[d]Assay that had a single incidence of homoplasy (Hornstra et al, 2011).

[e]Due to additional nucleotide differences in the allele-specific probes, this assay does not amplify samples that are ST21.

Supplemental Table 2. Primer information for constructing self-reporting synthetic positive controls for seven *Coxiella burnetii* TaqMan® dual-probe assays.

Assay Name[a]	Primer Sequences (5'→3')[b]	
Cox22bp91	AncFwd:	atcgactgtgttaggtcacTCGGGTGGTGCTCCCCTGTAGT
	DerFwd:	atcgactgtgttaggtcacTCGGGTGGTGCTCCCTTGTAGT
	Rev:	GCAAAAAGTGAGTGATAGCTGAGGAA
	PCR2 Fwd:	GCCTTCCATTATTGATTTATTGCCAAGAggatcgactgtgttaggtcac
Cox18bp376[c]	AncFwd:	atcgactgtgttaggtcacTGTTGGAATAACGAAGGCCAAA
	DerFwd:	atcgactgtgttaggtcacTGTTGGAATAACGAAGGTCAAA
	Rev:	GTCTCGGTTTAAATCAAGTAAGGGTAGT
	PCR2 Fwd:	CTTAAGTTGGCGCTTCTGTGATTggatcgactgtgttaggtcac
Cox51bp356[c]	AncFwd:	atcgactgtgttaggtcacTAAAAAGCTAGCGAAATTTGTTATTGC
	DerFwd:	atcgactgtgttaggtcacTAAAAAGCTAGCGAAATGTGTTATTGC
	[d]Rev:	ACCATTGCGAGCTTATCATCG
	PCR2 Fwd:	CGGAAATTTCACACGTATCTGCggatcgactgtgttaggtcac
Cox18bp166	AncFwd:	atcgactgtgttaggtcacATTGAAGCATACAAAACCTTCACGG
	DerFwd:	atcgactgtgttaggtcacATTGAAGCATACAAAACCTTCACGC
	Rev:	AGAGAGCGAGGTAAAGAGGCA
	PCR2 Fwd:	GCGAGGCCGTCTGCTGTAggatcgactgtgttaggtcac
Cox37bp215	AncFwd:	atcgactgtgttaggtcacCCTCTTTAAGTTTATCTGGGAG
	DerFwd:	atcgactgtgttaggtcacCCTCTTTAAGTTTATCTGGGAA
	Rev:	AGGAAATTTCTGGTTAAACCTTTCAAGGA
	PCR2 Fwd:	GCTGGGTGAAAAATTAATTCGGATTCAggatcgactgtgttaggtcac
Cox57bp327	AncFwd:	atcgactgtgttaggtcacAACAAGCTTTATTTGCCGACTTA
	DerFwd:	atcgactgtgttaggtcacAACAAGCTTTATTTGCCGACTTG
	[d]Rev:	GCTAATCAGTTAGTCAGATATCTTTAATTTT
	PCR2 Fwd:	ACGTTGGACTACTATTACTTGTCGGATAggatcgactgtgttaggtcac
Cox22bp118	AncFwd:	atcgactgtgttaggtcacGCGGAGAAAATATTGAACGACAA
	DerFwd:	atcgactgtgttaggtcacGCGGAGAAAATATTGAACGAAAA
	Rev:	GCGCTAAGCAAAAAGTGAGTGATAG
	PCR2 Fwd:	GCTGTCGGGTGGTGCTggatcgactgtgttaggtcac

[a]Assay/locus designation from Hornstra et al. (2011).
[b]AncFwd, sequence for the ancestral allele-specific forward primer for PCR1; DerFwd, sequence for the derived allele-specific forward primer for PCR1; Rev: sequence for the reverse primer for PCR1 and PCR2; PCR2 Fwd, sequence for the forward primer for PCR2. For all sequences: the target SNP-locus is shown in red text; excluding the SNP, upper-case letters match the native sequence of RSA493 (GenBank: AE016828.2); lower-case letters are non-native sequence to RSA493; blue text indicates the target site for the universal NED probe.
[c]PCR1 primers were designed from the compliment of the strand used to design the original probes for that assay.
[d]All reverse primer sequences, with the exception of those marked with a ([d]) are the same reverse primer used in the actual TaqMan® dual-probe assays listed in Supplemental Table 1 of this document.
